# Superwoman schema and metabolic syndrome in Black adolescent girls

**DOI:** 10.1007/s10865-025-00584-9

**Published:** 2025-07-09

**Authors:** Edith Chen, Jungwon Kim, Jayson Law, Vanessa Obi, Shanti U. Gallivan, Robin Hayen

**Affiliations:** 1 Northwestern University Institute for Policy Research, Evanston, IL 60208 USA; 2 Department of Psychology, 2029 Sheridan Rd., Northwestern University, Evanston, IL USA; 3 School of Law, University of Michigan, Ann Arbor, MI USA

**Keywords:** Metabolic syndrome, Stress, Coping, Adolescents, African American

## Abstract

This study investigated associations between the Superwoman schema (socialized expectations to project strength and exhibit a determination to succeed, while at the same time helping others and suppressing one’s emotions) and metabolic syndrome (MetS, a cluster of risk factors for diabetes, heart disease, and stroke detectable in childhood) across the period of adolescence. A sample of 256 Black adolescent girls (ages 14–19), all from lower-income households (≤ 2 × poverty threshold) was recruited for a cross-sectional study. Adolescents completed the Superwoman schema questionnaire, and MetS was measured using International Diabetes Federation criteria. Analyses posed a developmental question of whether associations varied by age across the period of adolescence. Age by Superwoman schema interactions were found, such that in younger adolescent girls, higher scores on the Superwoman schema questionnaire were associated with better cardiometabolic health (lower levels of MetS); however, by older adolescence, higher Superwoman schema scores were associated with worse cardiometabolic health (higher MetS). Psychologically, at older ages, a higher Superwoman schema score also was associated with experiencing greater conflict across life domains and with lower levels of perceived control. Overall these patterns suggest that a critical switch from the Superwoman schema being beneficial to being detrimental may occur some time during late adolescence. These findings suggest the importance of developing ways to cultivate and sustain the early beneficial aspects of a Superwoman schema as Black girls transition into adulthood.

## Introduction

The Superwoman schema is a construct that describes a coping approach that some Black women use to deal with the accumulation of stressors they experience as members of both subordinate racial and gender groups (Woods-Giscombe, 2010). It describes a strategy for maintaining toughness in the face of adversities—such as gendered racism—by projecting strength, exhibiting a determination to succeed along with a resistance to being vulnerable, and hiding one’s emotions, all while at the same time, being an important source of support and help to others. Many of these women describe a life of having to balance raising children and taking care of extended family members with working full-time and with their own educational aspirations, oftentimes having to handle responsibilities largely on their own, while experiencing skepticism from others about their ability to succeed. The perceived benefits of adopting a Superwoman schema have been described by Black women as survival-related—that is, seeking to preserve one’s sense of self-worth while also helping one’s family and one’s community to thrive (Woods-Giscombe, 2010; Sheffield-Abdullah & Woods-Giscombe, 2021).

The Superwoman schema allows Black women to rise above societally experienced discrimination and microaggressions through a combination of hard work, nurturance, and self-reliance. These traits are considered positive ones that are thought to be adaptive in many ways; however, at the same time, researchers have also proposed that these same traits could become detrimental over time, if both the effort required to constantly display strength, as well as the focus on helping others lead to less prioritizing of self-care and as well, to taking on the stressors of one’s social network (in addition to one’s own stressors) (Allen et al., 2019; Sheffield-Abdullah & Woods-Giscombe, 2021). After years of these experiences, Black women who evince a Superwoman schema are hypothesized to be at increased risk for stress-related health problems due to the mental and physical exhaustion of repeatedly giving to others and constantly trying to “do it all” without a break (Perez et al., 2023; Sheffield-Abdullah & Woods-Giscombe, 2021).

To measure the Superwoman schema, a questionnaire was previously devised and validated (Woods-Giscombe et al., 2019), that taps components (subscales) related to: (a) an obligation to manifest strength; (b) a determination to succeed; (c) a resistance to vulnerability; (d) suppression of one’s emotions; and (e) an obligation to help others, all of which can be combined into an overall Superwoman schema score. Consistent with the hypothesis that those who evince a Superwoman schema will be at increased risk for health-related problems are studies finding associations of this questionnaire with health behaviors such as sleep, diet, and physical activity. The Superwoman schema overall score has been associated with greater insomnia and all subscales also were associated with poorer sleep quality in Black women (McLaurin-Jones et al., 2021; Woods-Giscombe et al., 2019). The overall score also is associated with greater daytime sleepiness, and subscale analyses revealed that in particular, a greater obligation to help others is associated with poorer sleep quality, greater sleep disturbances, and greater daytime sleepiness in Black women (Erving et al., 2025). Other studies have found that the Superwoman schema is associated with stress-related eating; one study found that all Superwoman schema subscales were associated with a greater use of food as a means of coping with stress (Woods-Giscombe et al., 2019), whereas another study found that in particular, the subscales of obligation to help others and displaying resistance to vulnerability were associated with using food to cope with stress (Allen et al., 2019). Moreover, higher overall scores on the Superwoman schema questionnaire were associated with more stress-related emotional eating through lower levels of self-compassion and greater avoidance of negative thoughts and emotions (Volpe et al., 2024b; Volpe et al., 2024a). Higher overall scores on the Superwoman schema questionnaire also have been associated with lower levels of physical activity (Woods-Giscombe et al., 2019).

There are a small number of previous studies that have tested associations of the Superwoman schema with physical health outcomes or biological markers relevant to health. For example, the Superwoman schema subscale of an obligation to help others has been associated with poorer self-reported health in Black women (Erving et al., 2024a). The subscales of obligation to manifest strength and obligation to help others have been associated with a greater risk of hypertension in Black women (though motivation to succeed was associated with lower risk) (Perez et al., 2023). The resistance to vulnerability subscale has been associated with longer telomere length (repetitive DNA sequences that serve as caps on the ends of chromosomes preventing DNA degradation, considered a measure of biological aging; Thomas et al., 2022). And one study found interaction effects of a quadratic racial discrimination term with four of the five Superwoman schema subscales on allostatic load (cumulative wear-and-tear on physiological systems as a result of exposure to chronic stressors; Allen et al., 2019).

However, all of these previous studies have been conducted in adults, and hence little is known about associations of the Superwoman schema with health during earlier periods of life such as adolescence. The Superwoman schema has positive aspects, in the sense of being theorized to be a characteristic that promotes resilience and strength in the face of adversity. It is described as an asset and thought to be adaptive initially (Allen et al., 2019). Hence many Black adolescent girls may be encouraged to develop and exhibit Superwoman schema characteristics through racial socialization messages from their mothers related to showing strength (“be in charge of yourself”), striving for success (“make accomplishments on your own merit”), and helping others, as they are developing into young women (Thomas & King, 2007). To the extent that they are successful in their efforts, Black girls may initially experience positive reinforcements from others around them and develop a sense of pride and self-efficacy, and as a result, initially show better health. However, these same traits are posited to eventually become a liability over the course of years if they become overused and if they lead to weathering and exhaustion in the face of an accumulation of experiences with gendered racism (Allen et al., 2019). As these adolescents grow up and take on more adult responsibilities in their lives, they may be required to more frequently use their Superwoman schema to cope with accumulating burdens. Over time, then, this is proposed to become taxing and to lead to worse health, as has been observed in some instances in studies of adults (Perez et al., 2023; Allen et al., 2019). In the present study, we focus on the period of adolescence and hypothesize that associations of the Superwoman schema with health will vary by age, initially being associated with better health in younger adolescence, but then becoming associated with worse health in older adolescence.

The present study tested associations of the Superwoman schema questionnaire with an objective physical health outcome, metabolic syndrome (MetS), in a cross-sectional sample of Black adolescent girls, ages 14–19, all from lower-income households. MetS is a cluster of risk factors for diabetes, heart disease, and stroke later in life that is detectable in childhood and adolescence (Zimmet et al., 2007; Cornier et al., 2008). Hence MetS serves as a relevant physical health outcome and early warning sign of potential health problems in this relatively young and healthy sample. Our first hypothesis was that there would be an interaction of age with the Superwoman schema questionnaire, such that higher overall scores on the Superwoman schema questionnaire would be associated with better health (fewer indications of MetS) in younger adolescents, but with greater indications of MetS in older adolescents.

Psychologically, during the period of adolescence, responsibilities across life domains (e.g., school, home) increase for teens (Fuligni & Pedersen, 2002). Girls in particular are expected to provide greater assistance to the family (e.g., taking care of younger siblings, cooking) (Goodnow, 1988), and by adulthood, Black daughters are providing more help to parents than any other racial/gender group (Laditka & Laditka, 2001). Furthermore, adolescents from low-income families experience the greatest increases in family obligations during the teenage years (Fuligni & Pedersen, 2002). These adolescents experience many competing demands, as they often balance significant home, school, and work responsibilities (Chen et al., 2022), and the stress that adolescents experience in one domain spills over into other life domains (Flook & Fuligni, 2008). Thus for girls who adopt a Superwoman schema, in which they prioritize helping multiple others and handling a myriad of responsibilities on their own, creating unavoidable conflicts across domains, we hypothesize that the normative increases in daily life demands will lead to age by Superwoman schema interactions in conflict across life domains. That is, our second hypothesis in this cross-sectional study of Black girls from lower-income households was that associations between overall scores on the Superwoman schema questionnaire and conflict across life domains will become stronger at older ages.

As well, as these girls who are high in a Superwoman schema progress through adolescence and begin to balance increasing competing demands across multiple life domains, and in addition, become exposed to experiences with racism and sexism, they may develop perceptions related to a lack of control in their lives. In general, women, those from racial/ethnic minority groups, and those from lower-income backgrounds perceive less control in their lives (Lachman et al., 2011; Robinson & Lachman, 2017). In addition, greater experiences with discrimination are associated with lower levels of perceived control (Vargas et al., 2021). Thus our third hypothesis was that there would also be age by Superwoman schema interactions for perceived control, whereby associations between higher scores on the Superwoman schema questionnaire and lower levels of perceived control over one’s environment will become stronger at older ages. Finally, in exploratory analyses, we tested associations of the various subscales of the Superwoman Schema questionnaire and age with these same physical health and psychological outcomes.

## Method

### Participants and procedures

Participants were 256 Black girls, ages 14 to 19, drawn from a larger sample of 400 Black youth recruited for a broader study on life experiences and academic and health outcomes among lower-income Black youth (144 boys from the broader study were excluded in these analyses). Youth were recruited from the greater Chicago area through advertisements, presentations at schools, outreach to community organizations, and through a direct mail campaign. Eligibility criteria included youth who identified as Black, were between ages 14 and 19, whose family reported their income to be at or below two times the federal poverty threshold for their household size, being English speaking, having no current major chronic illnesses that necessitated taking regular medication, having no mental health disorder serious enough to warrant hospitalization in the past year, and having no pervasive developmental disorder that would make the youth unable to complete the study protocol. Participants who were currently pregnant or acutely ill were offered the option of rescheduling. Because the study hypotheses focused on Black girls, the analyses below included only female participants from the sample. We used sex at birth to categorize male/female because the MetS definition utilizes sex-specific criteria; in addition, 99.5% of participants in our sample identified their gender as the same as their sex at birth.

Eligible youth were invited for a laboratory visit, during which youth completed psychosocial questionnaires and health measures including a fasting blood draw (scheduled for the morning, generally between 8–10 am, to minimize diurnal variation). Youth provided either written assent or consent (depending on age), and caregivers provided written consent for all study procedures, which were approved by the Northwestern University Institutional Review Board.

### Measures

*Superwoman schema*. The original Superwoman Schema Questionnaire has 35 items (Woods-Giscombe et al., 2019; Sheffield-Abdullah & Woods-Giscombe, 2021). Because of time constraints related to the length of the protocol for the broader study, this questionnaire (as well as others) was shortened to 12 items, with 3 items chosen from each of the following subscales: obligation to manifest strength (“I have to be strong”); motivation to succeed (“I accomplish my goals with limited resources”); obligation to suppress emotions (“I keep my problems to myself to prevent burdening others”); and obligation to help others (“I put everyone else’s needs before mine”). In the shortened measure, efforts were made to represent the range of items, while eliminating items with redundancy. Resistance to vulnerability was dropped as a subscale in this study because of overlap of these questions (“It’s hard for me to accept help from others”) with other measures in the broader study related to asking for help specifically in the school context, and because previous studies did not identify resistance to vulnerability as one of the most salient components of the Superwoman role (Sheffield-Abdullah & Woods-Giscombe, 2021). Questions were answered on a 4 point scale, ranging from 1 = “This is not true for me” to 4 = “This is true for me all of the time.” Responses were averaged to create an overall Superwoman schema score, with higher numbers indicating greater endorsement of the Superwoman schema. Cronbach’s alpha for overall score using the shortened measure in the current study was 0.83, which is comparable to other articles that reported alphas for the overall measure of 0.80 and 0.88 using the original scale (Volpe et al., 2024b; McLaurin-Jones et al., 2021). Construct validity of the Superwoman Schema questionnaire in Black women was established in a previous article through associations with poorer mental health, sleep, and physical activity (Woods-Giscombe et al., 2019).

*Metabolic syndrome (MetS)*. Resting blood pressure was monitored with an automated auscultatory device (Carescape V100; GE) while the participant sat quietly. After a four minute acclimation, four readings were taken every 2 min, and the average of the last three readings was calculated. Waist circumference was measured at the midpoint of the upper iliac crest and lower costal margin, at the midaxillary line. Fasting blood was drawn into serum separator tubes. Serum was assayed at a local Core Laboratory for glucose and a cholesterol panel (visit times always scheduled in the morning to minimize diurnal variation).

MetS was diagnosed according to the International Diabetes Federation guidelines (Cornier et al., 2008). These criteria specify that the diagnosis of MetS requires central adiposity, which for the participants in this sample is defined as waist circumference ≥ 80 cm for females. At least two of four additional components must also be present. This includes: (a) signs of early hypertension (systolic pressure ≥ 130 or diastolic pressure ≥ 85 mm Hg), (b) elevated triglycerides (≥ 150 mg/dL), (c) elevated fasting glucose (≥ 100 mg/dL), or (d) lowered high-density lipoprotein levels (< 50 mg/dL in females).

Because of the young age of this sample, only 4% met criteria for MetS diagnosis. Hence we focused analyses on an outcome used in research with younger samples (Levine et al., 2019; Miller et al., 2018; Lam et al., 2024). This measure acknowledges concerns about the validity of dichotomizing youth into risk categories when metabolic functioning is distributed on a continuum (Goodman, 2008; Goodman et al., 2004). The measure entails creating a MetS composite by taking the z-score of each MetS component, and then averaging them, consistent with the approaches in the above previous studies.

*Perceived control.* This questionnaire assesses the extent to which individuals feel in control of their lives, and the extent to which they feel able to manage their environments and the demands of daily life. The Environmental mastery subscale of the Psychological Well-Being measure was used, which contains 3 items (e.g., “I am quite good at managing the many responsibilities of my daily life”) rated on a 6 point scale, from 1 = strongly disagree to 6 = strongly agree. This short version of the Psychological Well Being scale has been used in national studies and shows good psychometric properties (Ryff & Keyes, 1995). Note, however, that Cronbach’s alpha is relatively low, because of the small number of items (0.42 in the present study, 0.49 in the national study; Ryff & Keyes, 1995). Higher scores indicate feeling greater control over and more able to handle the responsibilities in one’s life.

*Conflict across life domains.* Youth were asked to make a rating of how much their school life and home life conflicted with each other (these being the two major life domains for most youth in our sample). For example, a participant could experience conflict if the pressures they experienced at school pulled them in different directions from the family obligations they had at home. Youth were shown a set of circles with varying degrees of overlap, and were asked to pick which set of circles best depicted this conflict, from “no conflict” (circles did not overlap) to “very conflicting” (circles had complete overlap). Circles were assigned a number from 1 to 9, with higher numbers depicting greater conflict. Twelve students did not complete this measure because of not being enrolled in school.

*Covariates*. Pubertal status and family socioeconomic status (SES) were included as covariates. Pubertal status was measured using a validated questionnaire, the Pubertal Development Scale (Petersen et al., 1988), which scores pubertal stage from 1 = pre-pubertal to 5 = post-pubertal. Family SES was calculated by standardizing and averaging caregiver reports of family income and family savings. Other demographic variables were not relevant to include as covariates in this study (age was a primary predictor variable, and the study consisted of all females and all participants from one race, Black).

### Statistical analyses

Study hypotheses were tested using linear regression equations with sequentially entered blocks of variables: (a) covariates of pubertal status and family SES; (b) age and Superwoman schema score; and (c) the two-way interaction term of age x Superwoman schema score. Outcome variables included MetS, conflict across life domains, and perceived control. Interaction analyses were conducted according to established guidelines, whereby variables were first mean centered and interactions were calculated as the product of the centered variables (Aiken & West, 1991). In exploratory analyses, the above analytic approach was repeated using each of the subscales of the Superwoman Schema. All statistical tests were 2-tailed with alpha set to 0.05.

## Results

### Preliminary analyses

See Table 1 for a description of the sample. Older age was correlated with higher Superwoman schema scores, r = 0.204, *p* =.001. Higher Superwoman schema scores also were correlated with lower perceived control over one’s environment, r = − 0.177, *p* = 0.005, and greater conflict across life domains, r = 0.205, *p* =.001. Lower perceived control also was associated with greater conflict across life domains, r = − 0.291, *p* <.001.

**Table 1:** Descriptive information and bivariate correlations (N = 256 girls)

	M	SD	1	2	3	4	5	6
1. Age	16.49	1.56						
2. Puberty	3.55	0.45	0.35**					
3. SES	0.00	0.77	0.09	0.11				
4. SS	3.11	0.52	0.20**	0.17**	−.01			
5. Control	4.31	0.82	− 0.09	0.02	− 0.02	− 0.18**		
6. Conflict	3.23	2.28	0.12	0.06	0.01	0.20**	− 0.29**	
7. MetS	− 0.09	0.54	0.04	0.02	0.00	− 0.01	− 0.05	− 0.09

Puberty, pubertal status. SES, socioeconomic status (standardized score of income & savings). SS, Superwoman Schema score. Control, perceived control. Conflict, conflict across life domains. MetS, metabolic syndrome (average of z-score of each component)

### Metabolic syndrome

**Fig. 1:**
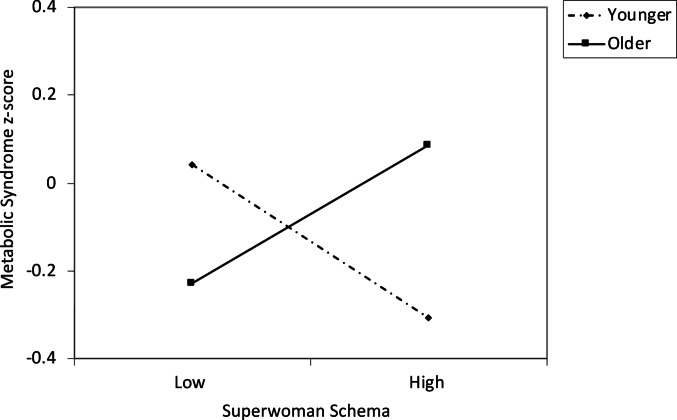
Metabolic syndrome as a function of scores on the Superwoman Schema questionnaire and age. The lines represent regression lines at different levels of participant age. Younger refers to 1.5 SD below the mean, effect size (standardized beta) = − 0.21. Older refers to 1.5 SD above the mean, effect size = 0.19. Metabolic syndrome z-score was calculated by standardizing each component of the metabolic syndrome and then averaging the standardized scores

There were no significant main effects of either age or Superwoman schema score on MetS. However there was a significant interaction effect, b = 0.091, se = 0.042, beta = 0.137, 95% CI [0.008, 0.173], *p* = 0.031, ΔR^2^ = 0.019. See Table 2. To interpret this finding visually, we plotted estimated levels of the MetS composite at low (1.5SD below the mean) and high (1.5SD above the mean) levels of age and the Superwoman schema score (Fig. 1). This graph reveals that at younger ages, higher scores on the Superwoman schema questionnaire are associated with lower levels of the MetS composite. In contrast, at older ages, the association shifts to becoming positive, such that higher scores on the Superwoman schema questionnaire are associated with higher levels of the MetS composite. Because our interest was in describing how patterns shift across the adolescent period, we did not test for the significance of any specific line on the graph, intending the figures more for illustrative purposes.

**Table 2:** Age and superwoman schema scores as predictors of metabolic syndrome and psychological experiences

	Beta	95% CI	*p*
Outcome: MetS			
SES	− 0.003	[− 0.090, 0.086]	.961
Pubertal status	0.018	[− 0.102, 0.136]	.780
Age	0.044	[− 0.031, 0.061]	.522
Superwoman schema	− 0.018	[− 0.151, 0.114]	.786
Age × Superwoman schema	0.137	[0.008, 0.173]	.031
Outcome: conflict across life domains			
SES	0.007	[− 0.353, 0.395]	.912
Pubertal status	0.043	[− 0.343, 0.684]	.513
Age	0.088	[− 0.066, 0.323]	.195
Superwoman schema	0.189	[0.272, 1.409]	.004
Age × Superwoman schema	0.121	[− 0.012, 0.687]	.058
Outcome: perceived control			
SES	− 0.027	[− 0.161, 0.103]	.666
Pubertal status	0.087	[− 0.054, 0.304]	.169
Age	− 0.095	[− 0.117, 0.019]	.153
Superwoman schema	− 0.182	[− 0.478, − 0.089]	.004
Age × Superwoman schema	− 0.121	[− 0.241, 0.000]	.050

MetS, metabolic syndrome; CI, confidence interval; SES, socioeconomic status

### Conflict across life domains

**Fig. 2:**
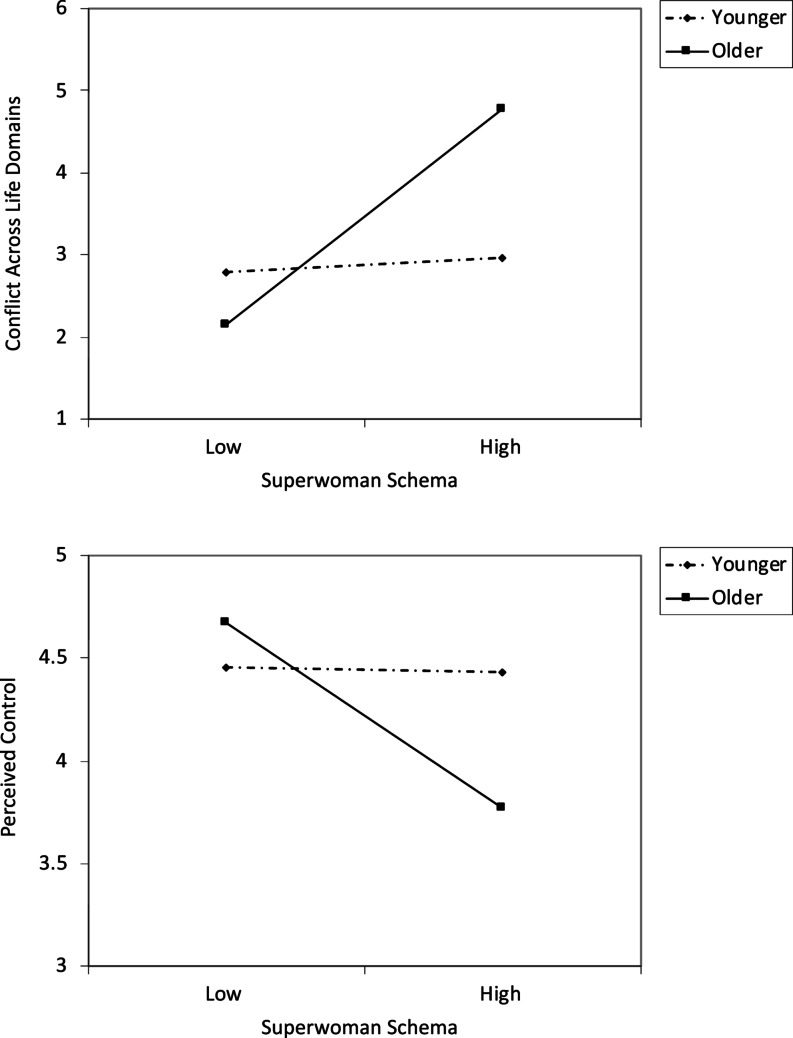
Conflict between school and home life (top panel) and perceived control over one’s life (bottom panel) as a function of Superwoman Schema score and age. The lines represent regression lines at different levels of participant age. Younger refers to 1.5 SD below the mean. Older refers to 1.5 SD above the mean. Effect size (standardized beta), conflict, younger = 0.02, older = 0.38. Effect size, perceived control, younger = − 0.01, older = − 0.37

There was no significant main effect of age on conflict across life domains. There was a significant main effect of the Superwoman schema score, indicating positive associations of Superwoman schema scores with conflict across life domains. However, this was qualified by a marginal interaction effect, b = 0.337, se = 0.177, beta = 0.121, 95% CI [− 0.012, 0.687], *p* = 0.058 ΔR^2^ = 0.014. See Table 2. Figure 2, top panel, depicts this interaction effect. This graph reveals that as youth get older, the association between Superwoman schema score and conflict becomes more positive, such that higher scores on the Superwoman schema questionnaire are associated with feeling more conflict between home and school.

### Perceived control

There was no significant main effect of age on perceived control scores. There was a significant main effect of Superwoman schema scores, indicating negative associations between scores on the Superwoman schema questionnaire and perceived control. However, this was qualified by an interaction effect, b = − 0.121, se = 0.061, beta = − 0.121, 95% CI [− 0.241, 0.000], *p* = 0.050, ΔR^2^ = 0.014. See Table 2. Figure 2, bottom panel, depicts the interaction effect. This graph reveals that as youth get older, the association between scores on the Superwoman schema questionnaire and perceived control becomes more negative, such that higher Superwoman schema scores are associated with feeling less in control of one’s environment and daily life.

### Exploratory subscale analyses

Analyses of the separate subscales of the Superwoman schema questionnaire revealed that the MetS interaction was driven by the obligation to manifest strength subscale (interaction effect: b = 0.076, se = 0.038, beta = 0.127, 95% CI [0.001, 0.150], *p* = 0.046, ΔR^2^ = 0.016), and the motivation to succeed subscale (interaction effect: b = 0.095, se = 0.034, beta = 0.174, 95% CI [0.028, 0.161], *p* = 0.006, ΔR^2^ = 0.030). Interactions with the other subscales were not significant.

In contrast, the conflict across life domains interaction was driven by the obligation to help others subscale (interaction effect: b = 0.273, se = 0.126, beta = 0.136, 95% CI [0.024, 0.521], *p* = 0.032, ΔR^2^ = 0.018). Interactions with the other subscales were not significant. The perceived control interaction was not driven by any particular subscale (interactions not significant for any subscale, indicating that it was the overall composite, or all subscales together, that contributed to this interaction effect).

## Discussion

The present study is the first that we are aware of to investigate associations between the Superwoman schema and both physical health and psychological outcomes in a sample of Black adolescent girls from lower-income households. During the teenage years, we found an interaction of the Superwoman schema questionnaire with age predicting metabolic syndrome (MetS). Younger teens showed a pattern of higher overall scores on the Superwoman schema questionnaire being associated with better cardiometabolic health (fewer indications of MetS), whereas older teens showed a pattern of higher Superwoman schema scores being associated with poorer cardiometabolic health (greater indications of MetS). This interaction was driven primarily by the subscales of an obligation to manifest strength in front of others and a determination to succeed in spite of limited resources. We also found an interaction of the Superwoman schema questionnaire and age with respect to psychological outcomes including experiencing conflict over life domains and perceptions of control over one’s life. With increasing age, the associations between higher overall scores on the Superwoman schema questionnaire and feeling greater conflict across life domains, and as well, feeling less able to manage (be in control of) the responsibilities in one’s life both became stronger. The interaction effect for conflicting life domains was driven by the subscale of obligations to help others. Overall, these results indicate that associations of the Superwoman schema scale with psychological and physical health outcomes vary throughout the period of adolescence in Black girls.

This study was cross-sectional, and findings will need to be replicated in studies with longitudinal designs. Nonetheless, the patterns are consistent with an explanation in which the Superwoman schema helps Black women cope with stress, but over time may begin to take a toll on Black women’s health (Allen et al., 2019; Perez et al., 2023). Because the traits of the Superwoman schema—being strong and driven to succeed, being inclined to help others—are generally considered positive ones, they may initially produce positive benefits for individuals who display them. Thus in younger Black adolescent girls, being higher on the Superwoman schema questionnaire was associated with fewer MetS indicators. However, the challenge may come after one has had to uphold a Superwoman schema consistently for a prolonged period of time. Given that with increasing age comes increasing numbers of life roles (Sumra & Schillaci, 2015), maintaining a Superwoman schema across years may begin to become exhausting and take a cumulative toll as these girls progress into adulthood. Thus we see that higher scores on the Superwoman schema questionnaire are associated with greater MetS indicators, as well as with feeling less in control of one’s life and feeling more conflict across life domains among older teens, consistent with patterns observed in mid-adulthood (Perez et al., 2023; Erving et al., 2024b; Woods-Giscombe et al., 2019). Overall these patterns suggest that a critical switch from the Superwoman schema being beneficial to being detrimental may occur some time during late adolescence.

In addition, certain Superwoman schema subscales seemed to be more relevant to certain outcomes. With respect to physical health, feeling an obligation to manifest strength and a determination to succeed were the subscales most strongly related to MetS. This is consistent with other literature on John Henryism (a characteristic involving persistent high-effort coping with external stressors) and skin-deep resilience (the theory that high striving and self-control will be beneficial to mental health but detrimental to physical health among groups exposed to high levels of adversity) that have documented associations of hard-driving work ethic, determination to succeed, high striving, and high self-control with outcomes such as increased risk for hypertension, diabetes, asthma, allostatic load, and metabolic syndrome in Black individuals, particularly from lower-income households (James et al., 1987; James et al., 1992; Brody et al., 2018; Brody et al., 2020; Chen et al., 2019; Brody et al., 2013). In fact, the present study extends previous research on skin-deep resilience showing associations of striving with poor physical health (Brody et al., 2016; Miller et al., 2016; Ehrlich et al., 2024) by providing evidence that adopting a Superwoman schema, in which one feels compelled to prioritize helping others over oneself and to juggle multiple responsibilities without displaying weakness or negative emotions, may be one factor that contributes to the poor health outcomes observed in skin-deep resilient youth, particularly Black adolescent girls from lower-income households. Taken together with the previous literature, these results suggest that it is a component related to grit, persistence, and trying to “do it all” constantly over a prolonged period of time that may be linked to poorer physical health outcomes in older Black adolescents, providing further support for theories such as skin-deep resilience (Chen et al., 2022).

In contrast, it was the subscale of an obligation to help others that was associated with the psychological experience of feeling greater conflict across life domains. This is consistent with previous research in Black women that has reported that an obligation to help others creates conflicts between taking care of oneself versus taking care of others (Sheffield-Abdullah & Woods-Giscombe, 2021), and leads to having to balance multiple simultaneous responsibilities and roles (Woods-Giscombe, 2010). In the present study, we saw the link between feeling an obligation to help others and feeling conflicted across life domains emerge more strongly among older teens.

It will be important to think through the implications of this study in terms of potential programs or interventions geared toward Black adolescents and women high in the Superwoman schema. There are many aspects of the Superwoman schema that are positive—for example, the desire to help others, and being highly motivated to succeed—so programs and interventions would not want to simply reduce these qualities in girls or women. And the fact that in younger adolescent girls, being high scorers on the Superwoman schema scale was not associated with poorer outcomes, either physically or psychosocially, suggests that perhaps there could be ways to cultivate the benefits of a Superwoman schema, without incurring the costs, as Black girls progress into adulthood. For example, this might entail finding ways to provide support to Black adolescents and young women (both tangibly and emotionally), so that they are not having to do as much on their own, and to help reduce the conflict for them across life domains. Indeed, research finds that people who experience more balanced levels of social support in their lives (in contrast to those who disproportionately give or receive support) have a lower risk of all-cause mortality (Chen et al., 2021). Similarly, non-reciprocity of support in close relationships is associated with poorer self-reported health and sleep problems, and those who focus on others to the exclusion of themselves (unmitigated communion) have worse metabolic control (Chandola et al., 2007; Helgeson et al., 2007). These findings suggest that finding the right sources and types of support may help Black older adolescents and young women high in the Superwoman schema to cope with the unique myriad of gendered/racial stressors in their lives. Researchers have also suggested that encouraging acts of self-compassion and self-care when confronting adversities and cultivating balance in one’s life may be fruitful avenues for future intervention research with this population (Volpe et al., 2024b; Joseph et al., 2023; Watson-Singleton et al., 2019). In addition, systems approaches that acknowledge the ways in which individuals are embedded within families, communities, and society more broadly, and that seek to make structural-level changes to mitigate the adversities experienced by individuals who are members of marginalized groups, and that acknowledge the ways in which the emergence of a Superwoman schema stems from these structural inequities, will be important to pursue in future research (Masten et al., 2021; Assari, 2018; Chen et al., 2024).

Limitations of the current study include the cross-sectional nature of the data, which precludes conclusions about causality or directionality. Future studies incorporating longitudinal designs will be necessary to track the development of these patterns across time and to determine whether these age-related changes occur within the same person over time. Lifecourse studies that investigate the Superwoman schema in Black girls and women across the lifespan would also be important. In addition, interventions to address the potential health costs of adopting a Superwoman schema would help address causality claims. Furthermore, expanding the types of outcomes investigated by age or life stage with respect to the Superwoman schema would be useful in future research. We note that another limitation of the present study was the low reliability of the perceived control scale, which suggests that findings related to perceived control in the present study should be interpreted with caution. Finally, future research should also investigate whether the Superwoman schema might apply to women from different racial/ethnic or socioeconomic groups.

In sum, the present study demonstrated in a sample of Black adolescents girls from lower-income households that there are age by Superwoman schema interactions during the period of adolescence. These interactions revealed that higher overall scores on the Superwoman schema questionnaire were associated with lower levels of MetS in younger adolescents, but with higher levels of MetS in older adolescents. In conjunction, with increasing age during the adolescent period, higher overall scores on the Superwoman schema questionnaire became associated with experiencing greater conflict across life domains as well as with lower perceived control. These findings suggest the importance of identifying more precisely the benefits of a Superwoman schema earlier in life in Black girls, and of better understanding how to potentially prolong those benefits so that as these girls grow up, Superwomen characteristics can continue to give women the opportunity to both prevail and thrive in our society.

## Data Availability

Because the participants who provided the data were informed that their study-related information would be kept confidential, the data for this study are not publicly available. The data can be available upon request with IRB approval and a data use agreement.
